# Implementation of Acupuncture in Routine Oncology Care: A Comparison of Physicians’, Nurses’, Physiotherapists’ and Acupuncturists’ Practice and Beliefs

**DOI:** 10.1177/15347354221132834

**Published:** 2022-11-18

**Authors:** Anna Efverman

**Affiliations:** 1 University of Gävle, Gävle, Sweden

**Keywords:** integrative medicine, nursing, integrative oncology, acupuncture, evidence-based care, attitudes, expectations, physiotherapy

## Abstract

**Background:**

It is important to investigate beliefs in acupuncture in professionals because professionals’ expectations may affect treatment outcomes.

**Aim:**

To document the type, number, and education of professionals practicing acupuncture. Further, to compare beliefs about the effectiveness of acupuncture for common cancer related symptoms in the different types of professionals.

**Methods:**

This cross-sectional study employed a questionnaire on practice and beliefs regarding acupuncture effects for symptoms that commonly occur in patients treated within oncology care settings. The respondents (n = 555) consisted of oncology professionals that is, physicians (n = 133), nurses (n = 172), and physiotherapists (n = 117). Additional respondents consisted of acupuncturists (n = 133), working outside approved health care.

**Results:**

Of the respondents, acupuncture was practiced by 4% of the physicians, 6% of the nurses, 58% of the physiotherapists, and 90% of the acupuncturists. The professionals believed acupuncture to be effective for pain (of the physicians, nurses, physiotherapists, and acupuncturists, 94%, 98%, 89%, and 99% respectively believed in the effectiveness), chemotherapy-induced nausea (corresponding figures: 74%, 89%, 89%, and 93%), and vasomotor symptoms (corresponding figures: 71%, 81%, 80%, and 97%). The physicians believed acupuncture to be effective in a mean of 5 symptoms, nurses in 6 symptoms, physiotherapists in 6 symptoms, and acupuncturists in 10 symptoms (*P* < .001).

**Conclusions:**

Since the professionals varied substantially regarding practice, education and beliefs in acupuncture, oncology clinics may consider delivering patient preferred acupuncture according to evidence-informed guidelines rather than on varying preferences among the professionals, since professionals’ treatment expectations may modify treatment outcomes.

## Introduction

In patients undergoing cancer therapy, 83% presented interest in receiving acupuncture for symptom reduction but only 1% received acupuncture.^1^ This gap between interest and implementation raises interest in studying the practice of and attitudes toward acupuncture among professionals. Integrative symptom management in oncology care is of broad interest since patients with cancer experience a variety of symptoms^2^ that acupuncture may relieve. Examples are pain,^3^ vasomotor symptoms due to iatrogenic menopause,^4^ and nausea and vomiting.^5^ Many patients ask for more than medical treatments to reduce burdensome symptoms.^6^ Sadly, some patients even reject cancer therapies because of their expected toll.^7^ Many patients regard non-pharmacological treatments as adjuncts to medications.^8^ Integrative oncology means patient-centered and evidence-informed cancer care, utilizing mind and body practices, natural products, or lifestyle modifications from different traditions, for example acupuncture, alongside conventional cancer therapies.^9^ Acupuncture seems integrated within western oncology care; more than half of 123 European oncology centers offer acupuncture.^10^ However, few patients interested in acupuncture received acupuncture for their symptoms.^1,6^

Use of and interest in the wide range of methods within integrative medicine have previously been described, both from the perspective of patients (n = 171^8^ patients and the perspective of oncology clinics (n = 123 clinics^10^). It seems important to investigate specifically the implementation and practice of acupuncture in oncology routine care, to be able to discuss whether beliefs are in line with the scientific evidence for acupuncture effects. Investigating practice and beliefs seems relevant since professionals’ treatment expectations highly affect the patients’ expectations and thus the treatment outcomes.^11^ Acupuncture-treated patients with low acupuncture treatment expectations were more likely to experience frequent stools compared to other patients during radiotherapy, irrespective of whether they received genuine or sham acupuncture in a previous randomized controlled study.^12^ In another study, acupuncture-treated patients who believed that they had lower risk than others to become nauseous during radiotherapy had significantly reduced risk for nausea.^13^ If health care professionals indicate negative attitudes, the attitudes may limit patients’ possibilities to communicate with the health care professionals^14^ regarding integrative medicine.^15-17^ A previous study presented that more than one-third of patients’ attempts to initiate discussion on complementary therapies were ignored by the oncologist.^15^ In contrast, shared decision-making between patients and health care professionals is widely recommended in delivering patient-preferred care. Consensus within the team of health care professionals in advice and recommendations regarding feasibility and effects of treatments appears to be important.^14^ If some professionals within the oncology health care team believe in the effect of acupuncture, they will probably communicate in a positive way regarding expected effects of acupuncture. Other professionals may have a more negative attitude. The conflicting messages may affect the patient’s treatment expectations,^11-13^ induce uncertainty and lower patient satisfaction in patients asking health care professionals for advice^15-17^ regarding acupuncture. Oncology physiotherapists generally believed in the effectiveness of acupuncture and practiced it for many symptoms occurring during cancer therapy^18^ while there is a lack of knowledge regarding other professionals’ belief in and practice of acupuncture in patients with cancer.

The aim of this study was to analyze implementation of acupuncture in routine oncology care by documenting the type, number, and education of professionals practicing acupuncture. A further aim was to compare attitudes in terms of beliefs about the effectiveness of acupuncture for common cancer-related symptoms in different types of professionals: physicians, nurses, physiotherapists, and acupuncturists.

## Methods and Materials

### Design and Setting

This descriptive cross-sectional cohort study includes Swedish professionals (n = 555) within (physicians, nurses, physiotherapists) and outside (acupuncturists) the oncology health care team. The study was performed in accordance with the declaration of Helsinki and the study did not require ethical approval because it did not involve sensitive personal information. This was specified in the Swedish law regulating ethical approval (SFS 2003:460) and confirmed in an advisory statement by the Ethical Review Board regarding data collections in health practitioners (Linköping, 2018/423-31). All participants gave informed consent. The Swedish health care system is mainly publicly financed, primarily through taxes levied by county councils, although private health care also exists. Out-of-pocket fees are low and regulated by law. Licensed health care professionals, practicing health care within in the tax financed “approved health care,” adhere to the Swedish health care law (2017:30). In 1984, the Swedish National Board on Health and Welfare approved acupuncture specifically for pain to be legally given by acupuncture-educated professionals within the “approved health care” system. In 1993, all other symptoms became legal to treat using acupuncture if there exists scientific evidence for its effects and the Swedish health care law (2017:30) was subsequently as applicable for acupuncture as for all other treatments within approved health care. All individual oncology professionals thus estimate their own competence and make their individual acupuncture treatment decisions, commonly based on papers reviewing the scientific evidence for effects of acupuncture in cancer related symptoms.^3-5,13,19-21^ Acupuncturists (ie, not licensed health care professionals) practicing acupuncture within private alternatives have no scientific evidence-based restrictions; they work outside the publicly financed “approved health care.” However, they are prevented from using acupuncture to cure cancer per se, and to treat children (<12 years).

### Sample

**Figure 1.**
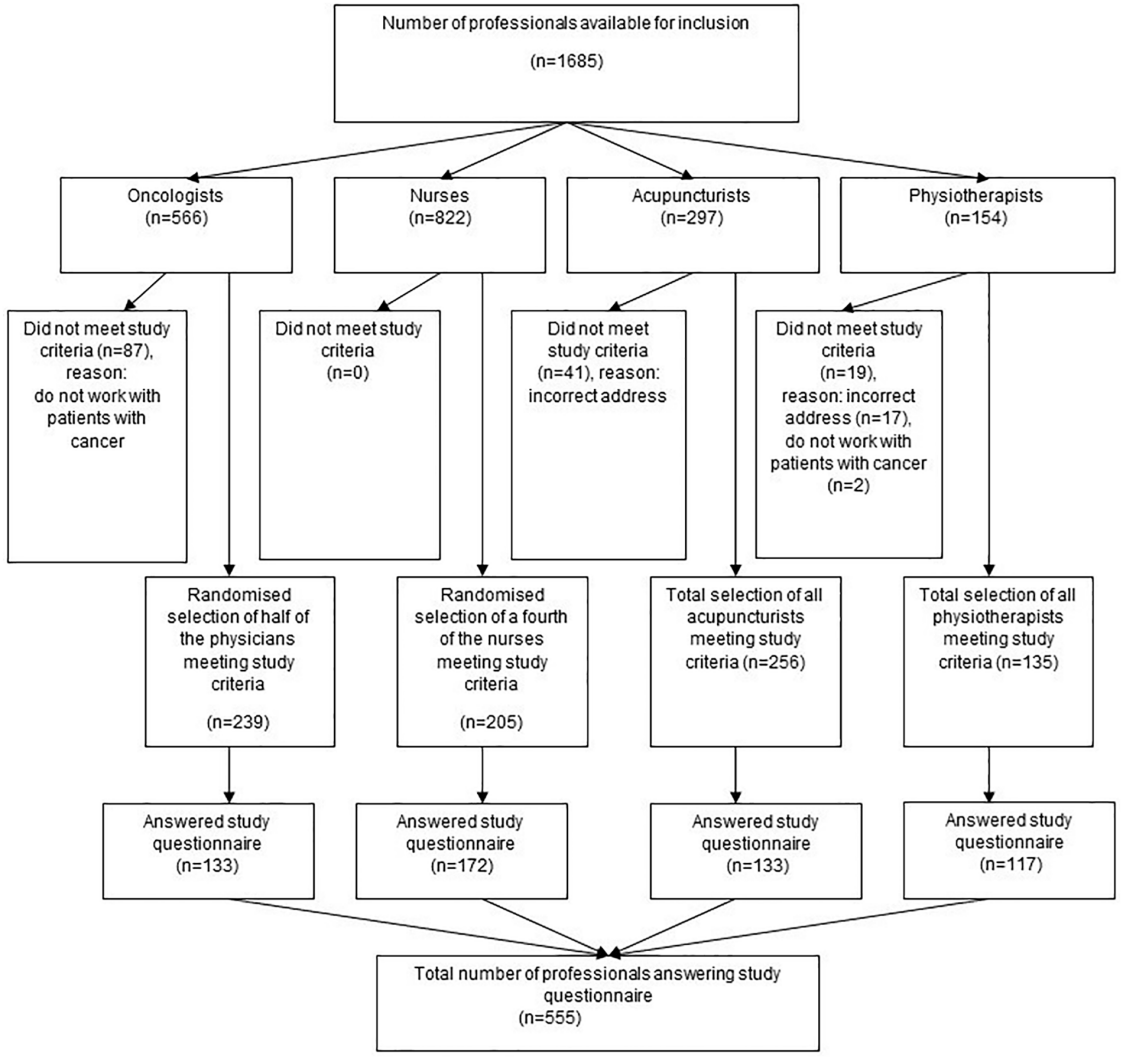
Flow-chart of the inclusion and data collection in the professionals.

With permission from the Swedish national organizations “Swedish Society of Oncology,” “Nurses in Cancer Care,” “Section for Oncology and Palliative Physiotherapy” and “Swedish Acupuncture Association Traditional Chinese Medicine” the study coordinators posted a letter comprising information about the study to the members and invited them to participate. The members of the organizations live and work all over Sweden. To obtain equal sized groups, a statistician had randomly selected half of the oncology physicians (n = 239), a fourth of the nurses (n = 205), all physiotherapists (n = 135) (previously presented^18^), and all acupuncturists (n = 256), by use of a computerized random-numbers table. The invitation letter declared that study participation was voluntary and confidential. Inclusion criteria were: professionals working with patients with cancer, or acupuncturists, and having a postal address in the member registers (Figure 1).

### Data Collection

Data were collected using a study-specific pre-tested and previously used^18^ questionnaire for professionals (Appendix), to be answered once. The questionnaire had an initial query confirming that the professionals worked with patients with cancer. If they did not (eg, were retired), they were excluded according to the study criteria. The questionnaire (Appendix) covered demographics (age, gender) and other descriptive variables (type of profession, number of years working in the profession, acupuncture education). Further, it covered questions regarding the professionals’ practice of acupuncture in patients with cancer and their belief in the effectiveness of acupuncture by answering the following “yes/no” question for a variety of symptoms commonly occurring in patients with cancer: “In what symptoms do you believe that acupuncture is effective for patients with cancer?” (cancer pain, edema, xerostomia, vasomotor symptoms, anxiety, fatigue, and nausea induced by chemotherapy, radiotherapy, morphine or surgery). The questionnaire asked the professionals to estimate the relevance of acupuncture: “In how large a proportion of your cancer patients would acupuncture be a relevant treatment? (7-grade scale; “none” to “all” of the patients). The questionnaire was confidential by use of a code replacing the name. It was posted to the study-coordinator using pre-stamped envelopes. If the professionals did not answer the questionnaire within approximately 2 weeks, a reminder was made by postal mail.

### Statistical Methods

The study evaluator calculated descriptive statistics for all variables, that is, number (n), percent (%), mean (m), standard deviation (SD) for continuous variables, median (md), as well as 25th and 75th percentiles for ordinal variables. The study evaluator summed the total number of symptoms for which each professional believed acupuncture to be effective: “number of symptoms.” The Kruskal-Wallis test compared physicians, nurses, physiotherapists, and acupuncturists regarding the continuous, not normally distributed, variable “number of symptoms” and post-hoc testing was made using Mann Whitney U-test. The evaluator compared the proportion of physicians, nurses, physiotherapists, and acupuncturists who believed acupuncture to be effective (stating “yes”) for the variety of symptoms using Chi-squared test. The Statistical Package for the Social Sciences (IBM SPSS Statistics for Windows, version 23, Armonk, NY: IBM Corp) was used, and the significance level was *P* < .05.

## Results

### The Professionals

Of the 1685 organization members, 1538 fulfilled the study criteria while 147 did not. After the randomized selection, 835 were asked to give their informed consent and 555 participated (total response rate 66%; physicians 56%; nurses 84%; physiotherapists 87%; acupuncturists 52%, Figure 1). A “typical” professional participating in the study was a woman (78%), 50 years old (ie, mean value), who had worked in his/her profession for 20 years (ie, mean value, Table 1).

**Table 1.** Demographics of the Professionals.

Variables	Total sample (n = 555)	Physicians (n = 133)	Nurses (n = 172)	Physiotherapists (n = 117)	Acupuncturists (n = 133)
Sex, n (%)					
Man	121 (22)	66 (50)	7 (4)	5 (4)	43 (32)
Woman	434 (78)	67 (50)	165 (96)	112 (96)	90 (68)
Age in years	(n = 550)				(n = 127)
mean ± SD	50 ± 10	51 ± 11	48 ± 8	48 ± 10	52 ± 10
Number of years working in the profession		(n = 132)	(n = 170)		(n = 128)
mean ± SD	20 ± 10	23 ± 11	20 ± 9	21 ± 11	14 ± 8

Numbers (n) and proportions (%) of professionals delivering data are presented, number delivering data is presented in case of missing data. SD = 1 Standard Deviation.

### The Type, Number, and Education of Professionals Practicing Acupuncture

Of the 541 of 555 professionals answering the question, 218 (40%) had an education in acupuncture therapy. Of the 530 professionals answering the question, 194 (37%) practiced acupuncture in patients with cancer. Among the oncology professionals, physicians (4%) and nurses (6%) were least likely to practice acupuncture, while physiotherapists were more likely to practice acupuncture (56%) (*P* < .001). Of the acupuncturists, 90% were practicing acupuncture.

Among the oncology professionals, physicians (2%) and nurses (8%) were least likely to be educated in performing acupuncture, while physiotherapists were more likely to be educated in performing acupuncture (65%) (*P* < .001). Of the acupuncturists, 97% were educated in acupuncture. Two of the physicians and 4 of the acupuncturists practiced acupuncture without having any acupuncture education (Table 2). Among the acupuncture-educated professionals, nurses were least likely to practice acupuncture (64%), while physiotherapists (90%), acupuncturists (91%), and physicians (100%) were more likely to practice acupuncture in patients with cancer (*P* < 001).

**Table 2.** Education In and Practice of Acupuncture.

Variable	Total sample (n = 555)	Physicians (n = 133)	Nurses (n = 172)	Physiotherapists (n = 117)	Acupuncturists (n = 133)
Are you educated in performing acupuncture therapy?^1^ n (%)	(n = 541)	(n = 129)	(n = 166)	(n = 117)	(n = 129)
Yes	218 (40)	3 (2)	14 (8)	76 (65)	125 (97)
No	323 (60)	126 (98)	152 (92)	41 (35)	4 (3)
Are you practicing acupuncture within your profession? n (%)	(n = 530)	(n = 124)	(n = 163)		(n = 126)
Yes	194 (37)	5 (4)	9 (6)	66 (56)	114 (90)
No	336 (63)	119 (96)	154 (94)	51 (44)	12 (10)

Numbers (n) and proportions (%) of professionals delivering data are presented, number delivering data is presented in case of missing data.

1 The question did not specify type or length of the acupuncture education.

Of the 320 professionals answering the question, the physicians were least likely to state that acupuncture would be relevant for half or more of patients with cancer (8%), while the physiotherapists (40%), nurses (48%) and acupuncturists (68%) were more likely to state this (*P* < .001, Table 3).

**Table 3.** The Professionals’ Estimation of the Proportion of Patients for Whom Acupuncture Would be a Relevant Treatment.

Proportion of patients for whom acupuncture treatment would be a relevant treatment Number (%)	Total sample (n = 320)	Physicians (n = 98)	Nurses (n = 97)	Physiotherapists (n = 103)	Acupuncturists (n = 22)
No patients	8 (3)	3 (3)	1 (1)	2 (2)	2 (9)
Few patients	117 (37)	60 (61)	20 (21)	34 (33)	3 (14)
Less than half	83 (26)	27 (28)	29 (30)	25 (24)	2 (9)
Approximately half	60 (19)	6 (6)	26 (27)	26 (25)	2 (9)
More than half	22 (7)	2 (2)	11 (11)	9 (9)	0 (0)
Most patients	19 (6)	0 (0)	9 (9)	6 (6)	4 (18)
All patients	11 (3)	0 (0)	1 (1)	1 (1)	9 (41)
Median 25th percentile, 75th percentile	Less than half, Less than half, Approximately half	Few, Few, Less than half	Less than half, Less than half, Approximately half	Less than half, Few, Approximately half	Most, Less than half, All

n, number of professionals delivering data was in total 320, since the other professionals stated “not a relevant question, I cannot answer” or “not a relevant question, I seldom meet patients with cancer.”

### The Attitudes to the Effectiveness of Acupuncture

**Figure 2.**
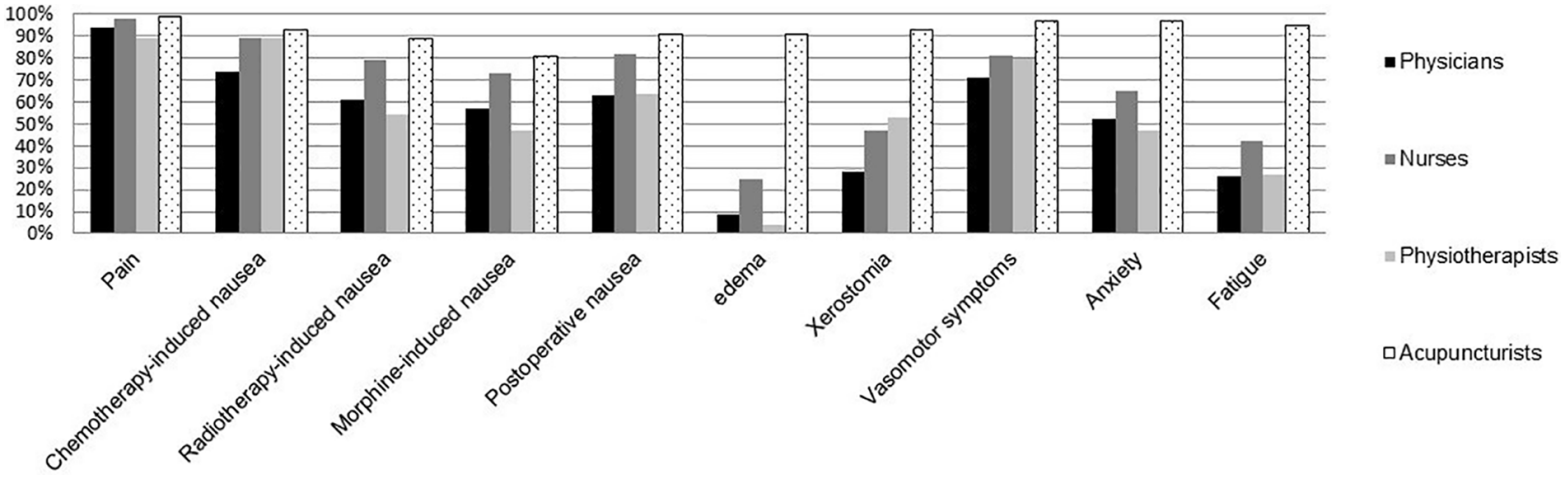
Percentages of surveyed professionals believing acupuncture to be effective for a variety of different symptoms in patients with cancer. Numbers delivering data were: 121 physicians, 160 nurses, 117 physiotherapists, 114 acupuncturists.

There were significant differences between professions in beliefs about the effectiveness of acupuncture for treating the variety of symptoms asked for (*P* < .001 regarding these 9 symptoms), except cancer pain (*P* = .106). Most professionals, irrespective of profession, considered acupuncture to be effective for cancer pain. The acupuncturists and the nurses were more likely than the other professionals to consider acupuncture effective for each of the 10 exemplified symptoms (Figure 2). For cancer pain (89%), radiotherapy-induced nausea (54%), morphine-induced nausea (47%), edema (4%) and anxiety (47%), the belief in the effects of acupuncture was lowest among the physiotherapists, while the corresponding figures were higher for the nurses (98%, 79%, 73%, 25%, and 65%). For chemotherapy-induced nausea (74%) and vasomotor symptoms (63%), the belief in the effectiveness of acupuncture was lowest among the physicians, while the corresponding figures were higher for the nurses (89% and 81%). Almost all professionals, except the acupuncturists and a fifth of the nurses, deemed acupuncture to be ineffective for edema. Accordingly, the professionals varied regarding how many symptoms they considered acupuncture to be an effective therapy for (*P* < .001). The physicians believed acupuncture to be effective in a mean of 5 symptoms (SD ± 2.7), nurses mean of 6 symptoms (SD ± 2.5), physiotherapists mean of 6 symptoms (SD ± 2.8), and acupuncturists mean of 10 symptoms (SD ± 2.0), which was all the symptoms asked for. Accordingly, the post-hoc testing revealed that the acupuncturists believed acupuncture to be effective in more symptoms than did the physicians (*P* < .001), the nurses (*P* = .007), and the physiotherapists (*P* < .001). The physicians did not differ from the nurses (*P* = .343) or the physiotherapists (*P* = .684). The nurses did not differ from the physiotherapists (*P* = .177).

## Discussion

This study found that almost all acupuncturists and more than half of physiotherapists practiced acupuncture, while few of physicians and nurses practiced acupuncture in oncology care. All the acupuncture-practising physiotherapists and nurses and almost all the acupuncturists were educated in acupuncture, while both the acupuncture-practicing physicians were acupuncture-uneducated. Two thirds of the educated nurses and 9 of 10 of the educated physiotherapists and acupuncturists practiced acupuncture. The professionals highly varied in their attitudes regarding their belief on efficacy of acupuncture for a variety of cancer related symptoms, but they highly believed acupuncture to be effective for cancer pain, nausea, and vasomotor symptoms. The acupuncturists, working outside the approved oncology health care, believed acupuncture to be effective in all 10 symptoms asked for, while oncology physicians, nurses and physiotherapists considered acupuncture to be effective in 5 to 6 common cancer related symptoms.

Regarding the observations of the type, number, and education of professionals practicing acupuncture, it was surprising that all physicians who practiced acupuncture did so without any acupuncture education. Swedish health care law (2017:30) requires health care practitioners to have skills in all tasks that they deliver, irrespective of type of task; no special regulations regarding acupuncture education are in use since 1993. The physicians may surprisingly have thought that they had enough skills in acupuncture to perform safe acupuncture treatments even without acupuncture education. Alternatively, they may have misinterpreted or ignored the Swedish health care law. The law requires a particular individual responsibility to deliver evidence based and safe care. Overall, integrative medicine should be evidence-informed.^9^

Great differences in attitudes to the efficacy of acupuncture were observed both within professions of the oncology team, for example, between physicians and nurses, and between professionals within the oncology team and acupuncturists working outside approved health care. The professionals most frequently believed acupuncture to be effective for cancer pain, nausea, and vasomotor symptoms. This is in line with the fact that the most common indications for acupuncture in 123 European oncology centers were nausea and vomiting (13% of all acupuncture treatments), pain (11%), vasomotor symptoms (11%), and fatigue (11%).^10^ The observation that a high proportion of the professionals believed acupuncture to be effective for pain was also in line with patients’ beliefs regarding acupuncture effects. Of n = 198^18^ and n = 457^1^ patients undergoing cancer therapy, 79% and 56%, respectively, believed acupuncture to be effective for pain. Seven randomized sham-controlled trials have demonstrated pain-reducing effects of genuine acupuncture in patients with cancer.^3^ Since psycho-neurobiological processes, for example, patients’ positive treatment expectations^11-13,23^ may activate descending pain inhibitory pathways^24^ it seems important that professionals communicate the expected positive pain-reducing effects to the patients.

Potential reasons for the great differences in attitudes to the efficacy of acupuncture within professions of the oncology team may be a result of the Swedish regulation of health care, acupuncture included. According to the regulation, all individual licensed professionals must estimate the efficacy of acupuncture to make their own evidence-based acupuncture treatment decisions. Plausibly their valuing is based on research on acupuncture for cancer-related symptoms.^3-5,13,19-21^ The conclusions to be drawn vary highly depending on whether the studies compare the effect of acupuncture to the effect of no acupuncture (often demonstrating great effects^22,23^), or if the comparison alternative is a sham device (often demonstrating minor or no effects^4,13^). Accordingly, it seems reasonable that differences in interpretation of scientific studies produce differences in beliefs within different professionals of the oncology team. The professionals probably vary in education and experience in critically reviewing scientific literature. Physicians and physiotherapists are used to making independent treatment decisions, while nurses more often adhere to physicians’ decisions. Further, previous acupuncture experiences might influence acupuncture attitudes. The acupuncturists, that is, not licensed professionals, have no scientific evidence-based restrictions. Their positive beliefs are probably based on positive experiences of having treated patients perceiving great effects of acupuncture. However, integrative oncology is both a patient-informed and an evidence-based field of cancer care.^9^ If more professionals within the oncology team will be up to date regarding integrative oncology, probably fewer patients would have to seek alternatives outside the tax-financed approved health care.

The professionals strongly believed acupuncture to be effective for nausea induced by a variety of cancer therapies. Electro-acupuncture of the traditional antiemetic point Pericardium 6 (PC6) on the wrist reduced vomiting more than sham electro-acupuncture performed with superficially inserted needles and standard care using older types of antiemetics (n = 104).^25^ Manual acupuncture did not reduce nausea more than sham, using a telescopic non-penetrating needle, during chemotherapy (n = 80)^26^ or radiotherapy (n = 215).^13,23^ During chemotherapy, manual acupuncture reduced nausea intensity and need for antiemetics compared to telescopic sham acupuncture or standard care (n = 68).^27^ Patients receiving acupuncture during chemotherapy (n = 70)^28^ or radiotherapy (n = 277)^23^ experienced less nausea compared to patients receiving standard care, including just antiemetics. Despite these conflicting results,^13,23,25-27^ reviews do not make separate conclusions regarding the antiemetic effect of acupuncture compared to credible sham-control and compared to standard care, respectively. Hypothetically, this may explain the observed differences in attitudes to antiemetic acupuncture across the oncology team in the current study.

The current study observed that 71% to 92% of the professionals believed acupuncture to be effective for vasomotor symptoms. However, when treating vasomotor symptoms in patients with breast cancer, manual acupuncture was not more effective than sham using a telescopic non-penetrating needle (n = 70).^29^ Acupuncture was more effective than sham using superficially inserted needles (n = 59, n = 94).^30,31^ In studies without sham control, the reduction of vasomotor symptoms of manual acupuncture was as great as using pharmacological treatment^32^ and greater than self-management (n = 190).^33^ In 120 patients with breast cancer, the mean reduction in hot flushes was greatest in patients receiving electro-acupuncture (reduction 7.4 steps on grading of hot flushes), followed by sham acupuncture (reduction 5.9 steps), pharmacological treatment using gabapentin (reduction 5.2 steps), and placebo pills (reduction 3.4 steps). Sham electro-acupuncture was accordingly more effective than genuine gabapentin.^34^ A literature review^4^ noted this pronounced placebo response. The conflicting results from studies using different kinds of control groups may have induced the conflicting attitudes to acupuncture effects across the oncology team.

The professionals’ attitudes may potentially affect estimations regarding safety of practicing acupuncture. The acupuncturists answered that they believed acupuncture to be effective for edema, while professionals within the oncology health care team did not. Penetrating the skin may increase the risk of edema in terms of surgery-induced lymphedema. The responding professionals might naturally have believed that this risk is valid also for specifically acupuncture needles. This supposed risk has no support from scientific studies.^35^ However, acupuncture did not reduce lymphedema more than control procedures did.^35^

Consensus within the health care team proved to be the strongest independent predictor of patient satisfaction, treatment acceptance, and adherence to treatments, in a study of 402 inpatients in general rehabilitation clinics.^14^ In the current study, there were instead great differences between the professionals regarding what proportion of the patients in oncology care they thought acupuncture treatment would be relevant for. Interestingly, less than 1 in 10 physicians stated that acupuncture would be relevant for half or more of patients with cancer, while nearly half of the physiotherapists, and more than half of the nurses and acupuncturists were more likely to state this. The physicians’ statement was in line with a previous study,^36^ presenting that only 7% of 1135 Norwegian physicians stated they could recommend a patient with cancer to receive acupuncture treatment. This kind of restrained assessment of the relevance of acupuncture in cancer care may potentially explain why just 1% of 457 Swedish patients stated that they have been treated by acupuncture for cancer-related symptoms even though 83% were interested in acupuncture treatment.^1^ Differences between professionals of the oncology health care team are problematic since the patients’ most important source of information on integrative medicine is the oncology professionals. Approximately 60% of patients who used complementary therapies asked for information from their oncology physicians and nurses.^17^ The health care professionals’ attitudes may affect whether patients dare to ask for advice on acupuncture. In a study of 755 Swedish cancer patients, just a third of 198 users of complementary therapies discussed this with their oncology professionals, mostly since they expected a negative attitude.^37^ The health care attitudes may affect what recommendations the patients receive from the professionals and accordingly what decisions the patients make during the shared decision-making procedure. The most significant determinant of oncology treatment decisions in general was recommendations from oncology physicians.^7,38^ Patients have expressed that they were more likely to develop positive preferences for acupuncture for cancer-related symptoms if they perceived the practice to be evidence-informed.^39^ The observed high variation regarding belief in the effectiveness of acupuncture among oncology professionals in the current study highlights the need for better methodological quality of future acupuncture studies. Designing better studies would facilitate development of appropriate guidelines for oncology professionals so that there will be fewer conflicting messages between professionals. The professionals’ beliefs regarding expected effects have been seen to modify the patients’ treatment expectations^40^ and the patients’ treatment expectancy in turn modified the effect of acupuncture in oncology care^12,13,41^ and other settings.^11^ In the light of previous findings presenting the acupuncture delivering therapist per se as a strong modifier of the effect, researchers may hypothesize that some of the variations in results between different acupuncture studies, regarding one and the same symptom, may be explained by the impact of the treating therapist.^40-42^

When the oncology professionals in the current study stated their beliefs regarding acupuncture effects, they might have considered that side-effects of acupuncture are mild and rather seldom occurring.^19,43^ If there are no better alternatives, acupuncture may be valuable to many patients despite the rather weak scientific evidence regarding genuine acupuncture effects.^3,9,20,21^ Although different studies of acupuncture for cancer-related symptoms present conflicting results^3-5^ and indicate great expectancy-driven effects,^3-5,12,13,23^ patients often present self-perceived benefits from acupuncture treatments for cancer related symptoms.^13,23,43^ Reasonably, effects from positive expectations, communication, touch, and relaxation during acupuncture therapy, may be valuable for the patients.^23^ Health care professionals always need to review the potential benefit and harm of their treatment options against each other in evidence-informed integrative medicine.^9,19^ Both the formal and the informal organization affect the decision-making regarding implementation of treatments in healthcare.^44^ To ensure that patients receive effective and safe integrative cancer care, including acupuncture, medical and nursing oncology knowledge as well as integrative cancer care competencies seem necessary. The findings of the current study highlight the need for integrative oncology education and training programs within the oncology team to meet the patients’ needs in integrative cancer care.^45^

A strength of this study is the number of respondents. Further, the study describes attitudes in a variety of professions that a patient with cancer may experience during the shared decision-making procedure regarding use of acupuncture, within and outside the oncology health team. The overall response rate of 66% may be considered as satisfactory when being compared to other studies on personnel, and the response rate of 84% of the nurses and 87% of the physiotherapists was even higher. The high compliance indicates that the development of the questionnaire through interviews made the study questions applicable to the respondents. A limitation is that only 320 of the 555 professionals delivered data regarding the estimated relevance of acupuncture. The non-responding professionals stated that this was not a relevant question. They did not want to answer since they seldom meet patients with cancer. The study was conducted in Sweden. Accordingly, the generalizability to countries with less publicly funded health care, or with specific national guidelines regarding acupuncture practice, is limited.

Since the professionals varied substantially regarding practice, education and beliefs in acupuncture, oncology clinics may consider delivering patient-preferred acupuncture according to evidence-informed guidelines rather than on varying preferences among the professionals, since professionals’ treatment expectations may modify treatment outcomes.

## Appendix

**Appendix.** Questions on Belief in and Implementation of Acupuncture.

Question	Type of scale	Lowest category	Highest category
In what symptom/symptoms do you believe that acupuncture is effective for patients with cancer?	Two-category nominal scale regarding 10 exemplified symptoms^1^	No	Yes
If you believe that acupuncture is effective in other symptoms, what symptom/s?	Free text responses, described using own words	-	-
Do you practice acupuncture treatment in patients with cancer?	Two category nominal scale	No	Yes
How often do you practice acupuncture treatments in patients with cancer?	Five-category ordinal scale	<1 treatment/week	Several treatments/day
For what symptom/symptoms do you practice acupuncture treatments in patients with cancer?	Two-category nominal scale regarding 10 exemplified symptoms^1^	No	Yes
If you practice acupuncture in other symptoms, what symptom/s?	Free text responses, described using own words	-	-
What proportion of the patients in oncology care would acupuncture treatment be relevant for?	Seven-category ordinal scale	None of the patients	All the patients
How large proportion of your patients with cancer receive acupuncture treatments performed by you or another therapist?	Seven-category ordinal scale	None of the patients	All the patients
If there are fewer who receive acupuncture than acupuncture would be a relevant treatment for, what is/are the reason/reasons? - Not relevant, all patients receive acupuncture if it is appropriate - Me or my colleges have no education in acupuncture therapy - Me or my colleges have not enough time for acupuncture treatments - Acupuncture is not included in my professional tasks - The patients’ medical treatments give not enough opportunity for acupuncture treatment - The patients’ are not interested in acupuncture	Two-category nominal scale	No	Yes

1 The professionals stated their belief in acupuncture effects for each of the 10 symptoms pain, xerostomia, vasomotor dysfunctions (hot flushes), anxiety, fatigue (extreme tiredness), edema and nausea induced by chemotherapy, radiotherapy, morphine, and surgery.
